# Comparison of Formulas for Low-Density Lipoprotein (LDL) Calculation for Predicting the Risk of Metabolic Syndrome

**DOI:** 10.31661/gmj.v9i0.1607

**Published:** 2020-01-27

**Authors:** Babak Pezeshki, Mojtaba Golrazeghi, Sayed Reza Hojati, Fatemeh Rostamian, Hadi Raeisi Shahraki, Mojtaba Farjam, Reza Homayounfar

**Affiliations:** ^1^Noncommunicable Diseases Research Center, Fasa University of Medical Sciences, Fasa, Iran; ^2^Student Research Committee, Fasa University of Medical Sciences, Fasa, Iran; ^3^Health Policy Research Center, Institute of Health, Shiraz University of Medical Science, Shiraz, Iran; ^4^Department of Epidemiology and Biostatistics, Faculty of Health, Shahrekord University of Medical Sciences, Shahrekord, Iran; ^5^Department of Nutrition, Fasa university of medical sciences, Fasa, Iran

**Keywords:** Cholesterol, LDL, Lipoproteins, Metabolic Syndrome, Friedwald

## Abstract

**Background:**

The correlation between serum cholesterol level and the risk of developing atherosclerosis and metabolic syndrome has been well established in previous studies. Serum low-density lipoprotein (LDL-C) measurement is conducted using different methods which are generally divided into two groups, namely direct and indirect. Using indirect methods or calculations such as the Friedewald or Iranian formula for measuring LDL, particularly in developing countries, is quite common. The present study has stepped in to compare the robustness of the extant formulas in prognosticating and determining the incidence of metabolic syndrome.

**Materials and Methods:**

In this cross-sectional study, the target population was the community of Fasa cohort study. According to the views of the statistical advisor, 9530 people were included in the study and clinical laboratory examinations were done for each person. Their serum LDL level was measured using the existing formulas. Then, the results of the serum LDL level that was computed with different formulas, were compared with both the status of metabolic syndrome and laboratory tests of individuals.

**Results:**

The Iranian formula has the highest area under curve, the sensitivity of 0.73, and specificity of 0.77, higher positive and negative predictive values among other formulas. In Friedewald formula, for example, sensitivity and specificity equal 0.28 and 0.80, respectively. After further analysis, two new models proposed for predicting metabolic syndrome. The results revealed that these two models even outperform the Iranian formula.

**Conclusion:**

The Iranian formula for plasma LDL calculation has higher precision and application for predicting and measuring the metabolic syndrome in the Iranian population due to its considerable features. It is required to develop a new formula for each population and even for each sex, if possible.

## Introduction

The correlation between serum cholesterol and the risk of developing atherosclerosis has been well established in previous research, such as Framingham study [1,2]. In human blood, much of the circulating cholesterol is carried by low-density lipoprotein (LDL); therefore, total cholesterol concentration is a good indicator of LDL cholesterol (LDL-C) level. According to the latest NCEP guidelines for the adult treatment panel, the diagnosis and treatment of hypercholesterolemia are entirely based on the measurement of total cholesterol and LDL-C levels [3]. Treatment aims to diminish LDL-C below the target values [2]. The LDL-C is a reliable marker for prognosticating the coronary heart disease. To date, various studies have asserted the strong correlation between increased LDL-C and coronary heart disease [4-6]. Serum LDL-C measurement is conducted using different methods which are generally divided into two groups, namely direct and indirect. There are various direct methods to this end; however, the reference and direct measurement of LDL is performed using a combination of ultracentrifugation-polianion precipitation, which is neither easily available nor feasible in routine labs. The new method for direct measurement of LDL is homogenous assay which is highly accurate, albeit considerably costly [7]. Considering the limitations mentioned above, using indirect methods or calculations such as the Friedewald formula for measuring LDL, particularly in developing countries, is quite common. However, evidence and some reports have revealed inconsistencies between the results of the homogeneous methods and those of the Friedewald formula, and this has led to a concerted effort to reach a more precise formula [8]. One of these attempts was made by Ahmadi *et al*. [9] who developed an Iranian formula for calculating LDL. Many other researchers, including Anandaraja [2], Vujovic [6], and Chen [10], also put forth new formulas for measuring LDL. Hitherto, no study, whether in Iran or abroad, has compared the robustness of all these formulas and their precision in predicting the status of metabolic syndrome in patients, hence the present study has stepped in to compare the robustness of the extant formulas in prognosticating and determining the incidence of metabolic syndrome.

## Materials and Methods

In Fasa Cohort Study, a part of the Persian cohort study, more than 10,000 people with age range of 35 to 70 years were investigated. The demographics, socioeconomic status, nutritional status, medical history, body composition, electrocardiogram test, and clinical laboratory examinations, were collected from each person. Also, a biobank of urine, blood, hair, and nail samples was compiled for further research. All information is recorded online for ease of access [11].

###  Exclusion and inclusion criteria

 All individuals with complete information were included in the study.

###  Research ethical

 Research committee registration code: IR.FUMS.REC.1397.096 In this cross-sectional study, the target population was the community of Fasa cohort study. According to the views of the statistical advisor, 9530 people were included in the study, and their LDL level was measured using the following formulas:

 Methods for measurement of LDL-C

1. **Fried Ewald’s formula [12]:**

 a. LDL-C (mg/dL) = TC-HDL-(TG/5)

 b. LDL-C (mmol/L) = TC-HDL-(TG/2.2)2

2. **Ananda raja’s formula (Indian) [2]:**

 LDL-C (mg/dL) = (0.9*TC)-0.9*(TG/5)-28

3. **Modified [6]:**

 LDL-C (mmol/L) = TC-(TG/3)-HDL

4. **Modified Fried Ewald’s formula [10]:**

 LDL-C (mg/dL) = Non-HDL*0.9-(TG*0.1)

 (Non-HDL=TC–HDL)

5. **A new accurate, simple formula [13]:**

 LDL = ¾ (TC - HDL)

6. **Iranian formula [9]:**

 a. LDL (mg/dL) = (TC/1.19)+(TG/1.9)-(HDL/1.1)-38

 b. LDL (mmol/L) = (TC/1.19)+(TG/0.81)-(HDL/1.1)-0.98

 In these formulas, TC, TG, and HDL represent total cholesterol, high-density lipoprotein, and triglycerides, respectively.

The metabolic syndrome is assumed to be present provided that three or more of the following parameters are met [14]:

 1. The waistline is more than 40 inches (102 centimeters) for men and more than 35 inches (89 centimeters) for women

 2. HDL is less than 40 mg/dL in men and less than 50 mg/dL in women

 3 Triglyceride level is 150 mg/dL or higher

 4. Blood pressure is 130/85 mm Hg or higher

 5. Fasting blood sugar is 100 mg/dL or higher

 Then, the results of the LDL level computed with different formulas were compared with both the status of metabolic syndrome and laboratory tests of individuals. After reviewing the results, more analysis was carried out to find more robust formulas..

###  Data analysis

 Descriptive statistics were reported as number (percentage) or mean± SD. Independent T-test was used to compare quantitative variables between two groups and logistic regression analysis performed for modeling associated factors with metabolic syndrome. Moreover, ROC curve analysis was used to obtain the area under the curve, sensitivity, and specificity of different LDL formulas. All the statistical analyses performed in SPSS (IBM Co., Armonk, NY, USA) 18.0 and MedCalc (Medcalc software, Ostend, Belgium) 14.0 software and P<0.05 considered as statistically significant.

## Results

**Figure 1:**
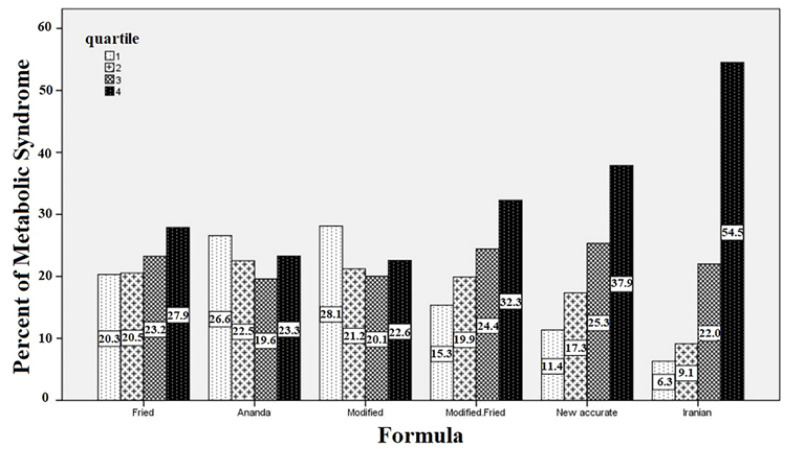
Proportion of metabolic syndrome among different quartile of each formula

**Figure 2:**
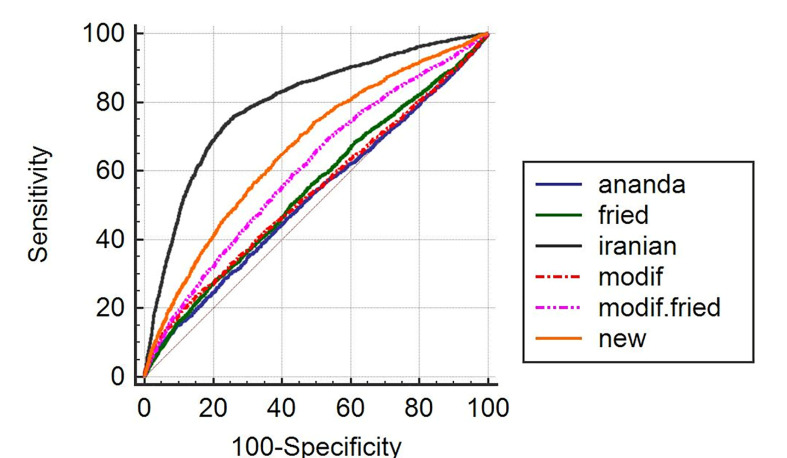
ROC curve of LDL formulas

Table-1 depicts the comparison of the variables under scrutiny between people with and without metabolic syndrome. The results confirm that except for HCT, SGOT, and RBC, other variables have significant differences between the two groups. Table-2 shows the LDL numbers calculated by different methods among people with metabolic syndrome and non-metabolic syndrome. Figure-1 exhibits the prevalence of the disease in each quartile of the formulas. For example, in the fourth quartile of the Iranian formula, 54.5% of people are afflicted with metabolic syndrome. The results of the Receiver Operating Characteristic (ROC) curve analysis are presented in Table-3. The columns represent the area under the curve (AUC), sensitivity, specificity, positive and negative predictive values, and optimum cut-off point, respectively. As observed, the Iranian formula has the highest area under the curve, sensitivity, and specificity. Figure-2 plots the formulas’ AUC. After further analysis, the results of two new models proposed for predicting metabolic syndrome are presented in Table-4. The results revealed that these two models even outperform the Iranian formula. The first model uses three variables, and the second model employs four variables. The AUC, sensitivity, and specificity of the models are reported at the end of the table, which portrays the superiority of the proposed models to all existing formulas. In the second model, the sex is also considered (in computations, one and zero stand for female and male, respectively).

**Figure:**
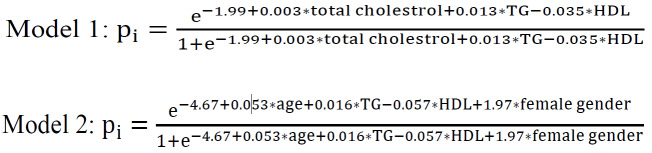


## Discussion

According to the results of the study, the Iranian formula for plasma LDL calculation has higher precision and application for predicting and measuring the metabolic syndrome in the Iranian population due to its considerable features, i.e., sensitivity of 0.73, specificity of 0.77, higher positive and negative predictive values, and area under the curve. In Friedewald formula, for example, sensitivity and specificity equal 0.28 and 0.80, respectively. In Table-4 that illustrates the first model, it can be observed that this model is more precise than the previous methods due to its sensitivity (0.76), specificity (0.77), and area under the curve (0.83). The second model, which also considers sex, is more robust than both the first model and other existing methods. According to the results presented in Table-1, metabolic syndrome has a significant correlation with most demographic indicators and laboratory data. The results exhibited in Figure-1 reveal that in Fried, Modified Fried, New Accurate, and Iranian formulas, more people with metabolic syndrome are present in the fourth quartile of the calculated LDL. Contrariwise, in Anandaraja and Modified formulas, the first quartile encompasses more afflicted people. Anandaraja *et al.* believed that the Friedewald formula shows variable percentages of agreement with the direct method in different geographic regions; therefore, they developed a new formula with a better agreement with direct measurement of LDL for Indian population compared with the Friedewald formula. Moreover, the LDL calculation by this method only requires triglyceride measurement, which is more cost-effective than the Friedewald formula [2]. De Cordova and colleagues in their study on a cohort of Brazil population found that the Friedewald formula fails to have a good agreement with direct measurement method for people with high or low triglyceride; thus, they proposed a formula that can be used to measure serum LDL in a wider range of populations with better agreement [13]. In their study, Vujovic *et al.* used Friedewald and Anandaraja formulas to calculate LDL of a Serb population and compared the results with direct LDL measurement. Then, they developed a new formula using the regression method to calculate LDL of the Serb population; they asserted that the proposed method is more accurate than the two formulas used in the study [6]. Chen *et al*. (2010) offered and tested a simple formula for calculating LDL. They compared their formula with Friedewald equation in terms of agreement with the direct method of measuring LDL and observed that the results of their new formula are much closer to the results of the direct method compared with the Friedewald formula. Additionally, their proposed formula dramatically lessens the interference generated by hypertriglyceridemia in computing LDL [10]. In another study, the Friedewald, Vujovic, Chen, and Anandaraja formulas were compared with eight direct methods of LDL computation. The findings indicated that for fasting samples in subjects with normal triglyceride (TG<200 mg/dL); the Friedewald formula has the best performance for measuring LDL. It should be noted that the precision of the Friedewald formula is variable depending on the HDL measurement method. None of the four formulas had good performance for samples with high triglyceride [15]. Likewise, Ahmadi *et al. *concluded that the Friedewald formula fails to be effective for people with high triglyceride level [9]. Mora *et al.* used the direct method and Friedewald formula to predict CVD from serum LDL-C in subjects with TG ≤ 400 mg/dL. The results showed that both methods are reliable for fasting samples [16]. In a study by Schectman *et al.*, it was shown that despite this widespread perception that the indirect formula, encompassing TC, TG, and HDL, may cause an error in the calculation of LDL or give an unrealistic estimation as a result of a change in a parameter and deviation from the normal range, the indirect method is not significantly different from the direct method [17].

## Conclusion

 Based on the results of the study, the Iranian formula for plasma LDL level calculation has higher precision and application and is the best one for predicting and measuring the metabolic syndrome in the Iranian population. As observed in the relevant studies, it is required to develop a new formula for each population and even for each sex, if possible, relying upon sufficient studies to reach the minimum error and maximum accuracy and performance.

## Acknowledgment

 The authors acknowledge Fasa University of Medical Sciences for financial supports of this paper (Project Code: 97036).

## Conflict of Interest

 The authors hereby affirm that the manuscript is original, that all statements asserted as facts are based on the accurate investigation, that the manuscript has not been published in total or in part previously and has not been submitted or considered for publication in total or in part elsewhere. Each author acknowledges he/she has participated in the work substantively and is prepared to take public responsibility for the work and authors have no competing interest in the results of this article.

**Table 1:** Comparison of Characteristics between Patients With IDF1 Metabolic Syndrome and Healthy Group

**Characteristic**	**IDF metabolic syndrome**		**P-value**
	**No (n=7322)**	**Yes (n=2186)**	**<0.001**
**Age**	48.21± 9.42	51.40± 9.23	<0.001
**BMI**	24.80± 4.62	28.66± 4.29	<0.001
**Waist circumference**	90.86± 11.33	101.22± 9.63	<0.001
**Hip circumference**	98.35± 8.68	103.61± 8.52	<0.001
**Wrist circumference**	16.60± 1.29	17.10± 1.43	<0.001
**WBC**	6.39± 1.73	6.76± 1.75	<0.001
**RBC**	4.96± 0.57	4.97± 0.57	0.51
**HGB**	14.74± 1.72	14.58± 1.67	<0.001
**HCT**	42.04± 4.43	41.85± 4.34	0.07
**MCV**	85.15± 7.84	84.57± 7.41	0.002
**MCH**	29.89± 3.30	29.48± 3.14	<0.001
**MCHC**	35.05± 1.24	34.81± 1.30	<0.001
**PLT**	270.48± 71.27	291.80± 74.43	<0.001
**LY**	42.40± 10.21	42.98± 9.93	0.02
**MO**	3.21± 1.36	3.31± 1.42	0.004
**GR**	54.37± 10.85	53.71± 10.62	0.01
**GLOC**	88.18± 20.20	109.02± 45.98	<0.001
**BUN**	13.03± 4.00	12.74± 3.86	0.003
**creatinine**	0.98± 0.20	0.97± 0.18	0.02
**TG**	113.02± 61.98	196.43± 106.69	<0.001
**Cholesterol**	182.63± 37.38	196.41± 42.45	<0.001
**SGOT**	22.47± 8.20	22.71± 8.93	0.25
**SGPT**	22.52± 13.74	25.91± 15.23	<0.001
**ALP**	206.49± 70.26	223.05± 66.37	<0.001
**HDL**	53.37± 16.35	45.05± 13.12	<0.001
**GGT**	21.52± 20.54	26.96± 20.91	<0.001

1. International Diabetes Federation

**Table 2:** Comparison of Obtained Scores in Each Formula between Cases with and without Metabolic Syndrome

**LDL Formula**	**IDF Metabolic Syndrome**		**P-value**
	**No**	**Yes**	
**Fried Ewald’s LDL**	106.65± 31.79	112.07± 35.67	<0.001
**Ananda raja’s LDL**	116.03± 31.17	113.41± 35.42	0.001
**Modified LDL**	91.59± 31.76	85.89± 39.38	<0.001
**Modified Fried Ewald’s LDL**	105.03± 29.68	116.58± 32.60	<0.001
**New accurate LDL**	96.94± 26.72	113.52± 30.14	<0.001
**Iranian LDL**	161.93± 71.35	251.16± 109.63	<0.001

**Table 3:** Results of ROC Analysis for Different Formulas

**LDL formula**	**AUC**	**Sensitivity**	**Specificity**	**PPV**	**NPV**	**Cut of point**
**Iranian formula**	0.80 (0.79-0.81)	0.73 (0.71-0.75)	0.77 (0.76-0.78)	0.49	0.91	196.35
**New accurate**	0.67 (0.66-0.68)	0.65 (0.63-0.67)	0.60 (0.59-0.61)	0.33	0.85	102
**Modified. Fried**	0.61 (0.60-0.62)	0.70 (0.68-0.72)	0.46 (0.45-0.47)	0.28	0.84	100.5
**Fried**	0.55 (0.54-0.56)	0.28 (0.26-0.30)	0.80 (0.79-0.81)	0.29	0.79	131.8
**Modified**	0.54 (0.53-0.55)	0.23 (0.22-0.25)	0.85 (0.84-0.86)	0.31	0.79	60.3
**Ananda**	0.52 (0.51-0.53)	0.15 (0.13-0.17)	0.91 (0.90-0.92)	0.33	0.78	77.3

**Table 4:** Results of Proposed Models to Predict Metabolic Syndrome

**Model 1**	**Model 2**
**Variable**	**OR (95% CI)**	**P-value**	**Variable**	**OR (95% CI)**	**P-value**
**Total cholesterol**	1.003 (1.002-1.005)	<0.001	**age**	1.05 (1.05-1.06)	<0.001
**HDL**	0.97 (0.96-0.97)	<0.001	**HDL**	0.945 (0.94-0.95)	<0.001
**TG**	1.013 (1.012-1.014)	<0.001	**TG**	1.016 (1.015-1.017)	<0.001
			**Female gender**	7.16 (6.20-8.26)	<0.001
AUC=0.83 (0.82-0.84), Sen=0.76, spec=0.77	AUC=0.87 (0.86-0.88), Sen=0.81, spec=0.78
